# The structure of the *TH/INS* locus and the parental allele expressed are not conserved between mammals

**DOI:** 10.1038/s41437-024-00689-y

**Published:** 2024-06-04

**Authors:** Trent Newman, Teruhito Ishihara, Geoff Shaw, Marilyn B. Renfree

**Affiliations:** 1 School of BioSciences, The University of Melbourne, Melbourne, VIC Australia; 2 Present Address: Epigenetics Programme, Babraham Institute, Cambridge, CB22 3AT UK

**Keywords:** Imprinting, Evolutionary genetics

## Abstract

Parent-of-origin-specific expression of imprinted genes is critical for successful mammalian growth and development. Insulin, coded by the *INS* gene, is an important growth factor expressed from the paternal allele in the yolk sac placenta of therian mammals. The tyrosine hydroxylase gene *TH* encodes an enzyme involved in dopamine synthesis. *TH* and *INS* are closely associated in most vertebrates, but the mouse orthologues, *Th* and *Ins2*, are separated by repeated DNA. In mice, *Th* is expressed from the maternal allele, but the parental origin of expression is not known for any other mammal so it is unclear whether the maternal expression observed in the mouse represents an evolutionary divergence or an ancestral condition. We compared the length of the DNA segment between *TH* and *INS* across species and show that separation of these genes occurred in the rodent lineage with an accumulation of repeated DNA. We found that the region containing *TH* and *INS* in the tammar wallaby produces at least five distinct RNA transcripts: *TH*, *TH-INS1*, *TH-INS2*, *lncINS* and *INS*. Using allele-specific expression analysis, we show that the *TH/INS* locus is expressed from the paternal allele in pre- and postnatal tammar wallaby tissues. Determining the imprinting pattern of *TH/INS* in other mammals might clarify if paternal expression is the ancestral condition which has been flipped to maternal expression in rodents by the accumulation of repeat sequences.

## Introduction

In one model of the evolution of genomic imprinting, increased maternal provision of resources to offspring in the mammalian lineage created a conflict between the parental genomes (Haig 2004; Smits et al. 2008). The placenta contributes to offspring development through several functions, including delivery of maternal nutrients and growth factors, and has been a focal point for investigating allelic gene expression under genomic conflict (Coan et al. 2005; Renfree et al. 2008; Bartolomei and Ferguson-Smith 2011; Tucci et al. 2019). Imprinting can also influence maternal investment through its function in postnatal tissues, such as the mammary gland and brain (Stringer et al. 2014; Cleaton et al. 2014; Tucci et al. 2019; Hanin and Ferguson-Smith 2020; John 2023). Relative to eutherians, the provision of resources to marsupial offspring is more pronounced during postnatal development but fewer imprinting studies have been made on these stages (Stringer et al. 2014; Hanin and Ferguson-Smith 2020). Parental conflict is entrenched in the literature as the primary selection pressure for genomic imprinting but other models could explain the evolution of imprinting for at least some genes (Spencer and Clark 2014).

Genomic imprinting has traditionally been studied in the fetus and the chorioallantoic placenta of mice and humans. The yolk sac evolved in fish while amniotes additionally developed the amnion, allantois and chorion. Mammals then adapted these four fetal membranes to support their *in utero* development (Mossman 1937; Ross and Boroviak 2020). Mammalian pregnancy depends on the yolk sac as the early site of maternal-fetal exchange, hematopoeiesis, and biosynthesis (Gulbis et al. 1998; Burton and Jauniaux 2021). In most marsupials it is the yolk sac that forms the definitive choriovitelline placenta and consists of an avascular bilaminar region (“BOM”: bilaminar omphalopleure) and a vascular trilaminar region (“TOM”: trilaminar omphalopleure), that in most species are closely apposed to the maternal uterine epithelium until birth (Renfree 1973, 2010; Freyer et al. 2003; Guernsey et al. 2017). In humans and mice the yolk sac supports the early stages of development before the establishment of the definitive chorioallantoic placenta (Ross and Boroviak 2020; Burton and Jauniaux 2023).

Imprinted genes are special cases, comprising only a small proportion of all mammalian genes. More than 200 imprinted “genes” are known in humans, including distinct transcripts expressed from the same locus, with the majority paternally expressed (Morison et al. 2005; Tucci et al. 2019). Slightly more imprinted genes are known in mice, with a quarter of these genes imprinted in both human and mouse (Tucci et al. 2019). So far, 25 autosomal genes showing parent-of-origin-specific gene expression have been found in marsupials (Smits et al. 2008; Stringer et al. 2014; Douglas et al. 2014; Ishihara et al. 2022; Cao et al. 2023; Bond et al. 2023). In marsupials, and the early extraembryonic tissues of some eutherians, X-inactivation is non-random with the paternal X chromosome imprinted to undergo silencing (Sharman 1971; Cooper et al. 1971; Grant et al. 2012; Lee and Bartolomei 2013). Marsupial imprinted X-inactivation is known to involve paternal expression of an *XIST*-like (X-Inactive Specific Transcript) noncoding RNA, *RSX* (RNA-on-the-Silent X), and maternal expression of a *TSIX* (“*XIST*” backwards)-like *RSX* antisense transcript, *XSR* (“*RSX*” backwards) (Grant et al. 2012; Mahadevaiah et al. 2020).

Imprinting control regions (ICRs) regulate parent-of-origin-specific expression of a neighbouring cluster of genes (Jacob 2013; Juan and Bartolomei 2019; Chang and Bartolomei 2020). The most ancient ICR known is ICR1 which is paternally methylated in both eutherians and marsupials and associated with maternal expression of the noncoding RNA, *H19*, and paternal expression of the insulin-like growth factor 2, *IGF2* (Sparago et al. 2004; Smits et al. 2008). A nearby ICR, ICR2, is located within the potassium voltage-gated channel gene, *KCNQ1*, and in eutherians has been associated with paternal expression of the *KCNQ1* overlapping transcript (*KCNQ1OT1*) and maternal expression of several other genes (Chiesa et al. 2012). ICR2 may not be present in marsupials because, although a *KCNQ1OT1* transcript is present in the TOM placenta of the tammar wallaby, the gene lacks a proximal CpG island (Ager et al. 2008). The genes that neighbour ICR2 in humans, mice and other eutherians, *CDKN1C* (cyclin dependent kinase inhibitor 1 C) and *PHLDA2* (pleckstrin homology-like domain family A member 2), are biallelically-expressed in the tammar wallaby (Suzuki et al. 2005, 2011).

Aberrant imprinting in the human chromosome 11p15.5 region, containing ICR1 and ICR2, is associated with the Beckwith–Wiedemann (BWS) and Silver–Russell (SRS) syndromes. BWS and SRS are clinically opposite growth disorders, birth weights for BWS are >90th percentile while in SRS they are <3rd percentile (Wollman et al. 1995; Weksberg et al. 2010; Jacob et al. 2013; Eggermann et al. 2008). BWS/SRS have a range of molecular subtypes, ICR2 hypomethylation is the most frequent (50–60%) in BWS, with ICR1 hypermethylation (5–10%) also found; ICR1 hypomethylation is the most frequent (50–60%) for SRS (Eggermann et al. 2008; Wang et al. 2020). Loss of methylation at the maternal ICR2 allele in BWS reduces expression of the growth regulator *CDKN1C* while loss of methylation at the paternal ICR1 allele in SRS increases *H19* which represses the growth promoting *IGF2* (Nativio et al. 2011; Naveh et al. 2021). Expression of the genes in the 11p15.5 region is developmentally important but how parent-of-origin-specific gene expression is regulated at the interface of the two ICRs is unknown.

The tyrosine hydroxylase (*TH*) / insulin (*INS*) locus adjoining ICR1 and ICR2 has a dynamic evolutionary history of gene duplication and loss. The ancestral amino acid hydroxylase genes and insulin-like genes became linked in the genome of early chordates (Patton et al. 1998; Yamamoto et al. 2010). Duplication of the insulin-like gene early in the vertebrate lineage resulted in the neighbouring *INS* and *IGF* genes that encode structurally similar proteins (Chan and Steiner 2000). The duplication event that resulted in *TH* and phenylalanine hydroxylase (*PAH*) predates the divergence of invertebrates (Patton et al. 1998). A further duplication event in jawed vertebrates resulted in one paralogy group containing *PAH, TH2* and *IGF1* and another containing *TH(1), INS* and *IGF2*, with the *TH2* gene subsequently lost in therians but not birds (Patton et al. 1998; Candy and Collet 2005; Yamamoto et al. 2010).

The insulin gene, *INS*, is an important regulator of carbohydrate metabolism that is paternally-expressed in the therian yolk sac (Ager et al. 2007). In humans, *INS* is monoallelically-expressed from the paternal allele in the yolk sac at weeks 9 and 10 of gestation (Moore et al. 2001) and there is circumstantial evidence for monoallelic expression in the thymus (Pugliese et al. 1997), neonatal pancreas and an adolescent spleen (Pugliese and Miceli 2002). Murine rodents, mice and rats, have two insulin-coding genes, *Ins1* and *Ins2*, with *Ins2* orthologous to the *INS* gene (Shiao et al. 2008). *Ins1* and *Ins2* are biallelically-expressed in mouse fetal pancreas and embryonic body, but only *Ins2* is expressed in the yolk sac (Giddings et al. 1994; Deltour et al. 1995, 2004). Both parental alleles of *Ins2* are expressed in the yolk sac at embryonic day E12.5, but by E14.5 the maternal allele is silenced so that *Ins2* is paternally-expressed in the yolk sac (Giddings et al. 1994; Deltour et al. 1995, 2004). Deletion of the ICR1 and *H19* region in mice results in activation of the *Ins2* maternal allele in the E13.5 yolk sac, indicating that ICR1 can regulate imprinted expression of *Ins2* in addition to *Igf2* and *H19* (Leighton et al. 1995).

The *INS* gene is paternally-expressed in the yolk sac and also in several postnatal tissues of the tammar wallaby (Stringer et al. 2012). *INS* has paternally-skewed expression in the BOM and TOM regions of the yolk sac placenta during the final third of the short, 26.5 day, tammar wallaby gestation after diapause (Ager et al. 2007). Marsupial mammals give birth to highly altricial young that are supported, usually in a pouch, by a sophisticated lactation process in which the composition of the milk changes dynamically throughout the whole of pouch life (Tyndale-Biscoe and Renfree 1987; Green et al. 1988; Trott et al. 2003; Stringer et al. 2014). Postnatally, *INS* is biallelically-expressed in the stomach and intestine, but monoallelically-expressed in the adult mammary gland and paternally-expressed in the tammar wallaby pouch young (PY) liver (Stringer et al. 2012).

The *TH* gene adjacent to *INS* encodes tyrosine hydroxylase: a key enzyme in the synthesis of catecholamines, particularly the neurotransmitter dopamine (Lelou et al. 2022). *Th* is maternally-expressed from E7.5 in the mouse placenta and embryo, but becomes biallelic within the embryo by E12.5 (Golding et al. 2011; Jones et al. 2011; Okae et al. 2012). In mice, the paternal *Th* allele is silenced in the E13.5 placenta but can be activated by deletion of ICR2 which prevents *Kcnq1ot1* transcription (Jones et al. 2011), indicating that *Th* is imprinted by ICR2 in the mouse. Whether *TH* has parent-of-origin-specific expression is not known in humans (Lefebvre 2012; Cleaton et al. 2014), or any other species.

Synteny of *TH* and *INS* is well conserved in vertebrates, with the exception that *INS* was lost from the *TH/IGF2* linkage group in some teleosts (Collet et al. 1998; Rotwein 2018). In chickens the distance between *TH* and *INS* is 15.5 kilobases (kb) while in humans the genes are 2.4 kb apart (Hernández-Sánchez et al. 2006). In contrast, the mouse orthologues, *Th* and *Ins2* are separated by a 210 kb region of repetitive DNA rich in retro-elements (Shirohzu et al. 2004). Chimaeric *TH-INS* transcripts, which fuse exons from both *TH* and *INS*, have been observed in the developing chicken, quail (Hernández-Sánchez et al. 2006; De Pablo and de la Rosa 2011) and tammar wallaby (Stringer et al. 2012, 2014). The chimaeric *TH-INS* proteins have lower enzymatic activity than *TH* but any roles for the chimaeric transcripts in regulating the expression of two adjacent genes, and any physiological implications, are unknown (Hernández-Sánchez et al. 2006).

Since insulin is a key hormone for growth and lactation in all mammals, we asked whether the large distance between *Th* and *Ins2* in the mouse was an ancestral mammalian trait or specific to mice. We examined a range of vertebrate species and found that the large distance between these two genes in mice is a feature specific to rodents. Despite the position of the *TH* and *INS* genes at the interface of ICR1 and ICR2, it is unknown if *TH* is expressed from the maternal allele in any other species, as in mice. This study investigated the orthologous region in the tammar wallaby to identify transcribed RNAs and analyse parent-of-origin-specific expression in tammar wallaby fetuses and PY. We identified five transcripts, *TH*, *TH-INS1*, *TH-INS2*, *lncINS* and *INS*, produced from the tammar wallaby region and show that each has paternal expression.

## Materials and methods

### Species comparison

The genomic location of *TH* and *INS* orthologues were taken from the 38 species listed in Supplementary Table 1. The distance between *TH* and *INS* was calculated as the end position of the *TH* gene minus the starting position of the *INS* gene, these values are provided in Supplementary Table 1. The species divergence times (mya) were taken from TimeTree 5 (Kumar et al. 2022), the divergence times relative to the house mouse are also noted in Supplementary Table 1. The phylogeny was visualised using the Environment for Tree Exploration: ETE v3 (Huerta-Cepas et al. 2016). Repeat sequences in the region between the rodent *Th* and *Ins(2)* genes were assessed using RepeatMasker v4.1.5 (Tarailo-Graovac and Chen 2009) with the “-species rodent” option.

The location of genes in the imprinted region was visualised for house mouse, human, cattle and tammar wallaby using the DNA Features Viewer library (Zulkower and Rosser 2020). ICR locations were from NCBI (Gene ID: 105317033, 105259599) or based on the position of published bisulfite primers (Smits et al. 2008; Oh et al. 2008; Robbins et al. 2012; Wang et al. 2015; Huang et al. 2021). The position of *KCNQ1OT1* in cattle and the tammar wallaby is from primer positions used to generate a short amplicon of the transcript (Ager et al. 2007; Robbins et al. 2012). Parent-of-origin-specific expression of genes in the region was from online databases https://www.geneimprint.com and https://corpapp.otago.ac.nz/gene-catalogue or literature, in the case of cattle *KCNQ1OT1* (Robbins et al. 2012; Chen et al. 2015).

### Animal samples

Tammar wallabies of Kangaroo Island, South Australia origin, were held in open grassy yards in our breeding colony at the University of Melbourne. A postnatal sample set was prepared containing tissues from 11 pouch young (PY) aged between 37 and 41 days postpartum (pp) and from 12 PY aged between day 78 and 81 pp, matched to maternal tissues. A prenatal sample set was prepared from the BOM and TOM placental tissues and the maternal endometrial tissues from 16 pregnant females and their fetuses collected between day 18 and 25 of the 26.5 day gestation. Tissue samples were collected as described previously (Suzuki et al. 2005; Ishihara et al. 2021) snap frozen in liquid nitrogen and stored at -80°C. After retrieval of the snap frozen PY whole brain tissue samples, the anterior portion of the brain including the olfactory bulb and the front half of the cerebrum was taken (Renfree et al. 1982) for RNA extraction. All tammar animal handling, husbandry and experimental sampling were in accordance with the National Health and Medical Research Council of Australia (2013) guidelines and approved by the University of Melbourne Animal Experimentation Ethics committees.

### lncRNA identification

To identify potential antisense lncRNA at the *TH/INS* locus, a publicly available tammar testis RNA-seq data set was analysed (NCBI, DRX012262). Reads were trimmed using TrimGalore! v0.6.10 (https://github.com/FelixKrueger/TrimGalore), aligned to the tammar wallaby genome v7 using HISAT2 v2.2.1 (Kim 2019) with the “--rna-strandness FR” option and mapped reads assigned to each strand with Samtools v1.16.1 (Li et al. 2009).

To determine the full-length of the antisense *INS* lncRNA transcript, RACE (rapid amplification of cDNA ends) was performed with the SMARTer RACE 5′/3′ kit (cat. no. 634923, Clontech, California, USA). The first round RACE reactions were performed with adult testis cDNA using SeqAmp DNA Polymerase (cat no. 638504, Clontech, California, USA) with gene specific primers (Supplementary Table 2). Nested RACE experiments were performed with GoTaq Master Mix (cat. no. M5123, Promega, Madison, WI, USA) and products cloned using the pGEM-T Easy Vector System (cat. no. A1360, Promega, Madison, WI, USA) and JM109 Competent Cells (cat. no. L2001, Promega, Madison, WI, USA). Plasmids were extracted using the Wizard Plus SV Minipreps DNA Purification System (cat. no. A1460, Promega, Wisconsin, USA) and sent for Sanger sequencing by the Australian Genome Research Facility (AGRF).

### Genotyping

Maternal and PY gDNA was prepared from frozen tissue using the Wizard Genomic DNA Purification Kit (cat. no. A1120, Promega, Madison, WI, USA) with a T10 basic handheld homogenizer (IKA, Staufen, Germany). Genotyping was carried out by PCR using the genotyping primers listed in Supplementary Table 2. PCR products were extracted using the QIAquick Gel Extraction Kit (cat. no. 28706, Qiagen, Venlo, Netherlands) and sent for Sanger sequencing by AGRF. Informative animals were those in which the offspring was heterozygous at a site for which the corresponding maternal sample was homozygous.

### RT-PCR

PY RNA was prepared from frozen tissue using the GenElute Mammalian Total RNA Miniprep Kit (cat. no. RTN70-1KT, Sigma-Aldrich, St. Louis, MO, USA). cDNA was prepared from 1 μg of RNA using the Superscript IV First-Strand Synthesis System (cat. no. 18091050, ThermoFisher Scientific, Waltham, MA, USA), primed with oligo(dT)_20_. Allele-specific expression analysis of each SNP was performed in multiple animals with a single technical replicate of RT-PCR using the expression primers listed (Supplementary Tables 2 and 3). PCR products were gel extracted using the QIAquick Gel Extraction Kit (cat. no. 28706, Qiagen, Venlo, Netherlands) and sent for Sanger sequencing by AGRF.

### Quantification of allele use

Parent-of-origin-specific gene expression was quantified by extracting the signal data from the .ab1 trace file. The signal intensity value for the major and minor alleles at an offspring SNP site was compared with reference to the identity of the genotyped maternal allele. The parental expression of the allele in the offspring cDNA was presented as a “mat:pat ratio”, the maternal signal / (maternal signal + paternal signal), metric which ranged from 0 to 1, where paternal expression was 0, biallelic expression was 0.5, and a value of 1 would indicate maternal expression. A mat:pat ratio of 0.00–0.20 or 0.80–1.00 was interpreted as “monoallelic”, 0.20–0.35 or 0.65–0.80 was interpreted as “skewed”, and 0.35–0.65 was interpreted as “biallelic”. The mean of multiple SNPs was taken and the final value provided is the mean ± standard error of the mean (SEM), across n animals.

## Results

### Separation of the *TH* and *INS* genes in the rodent lineage

**Fig. 1:**
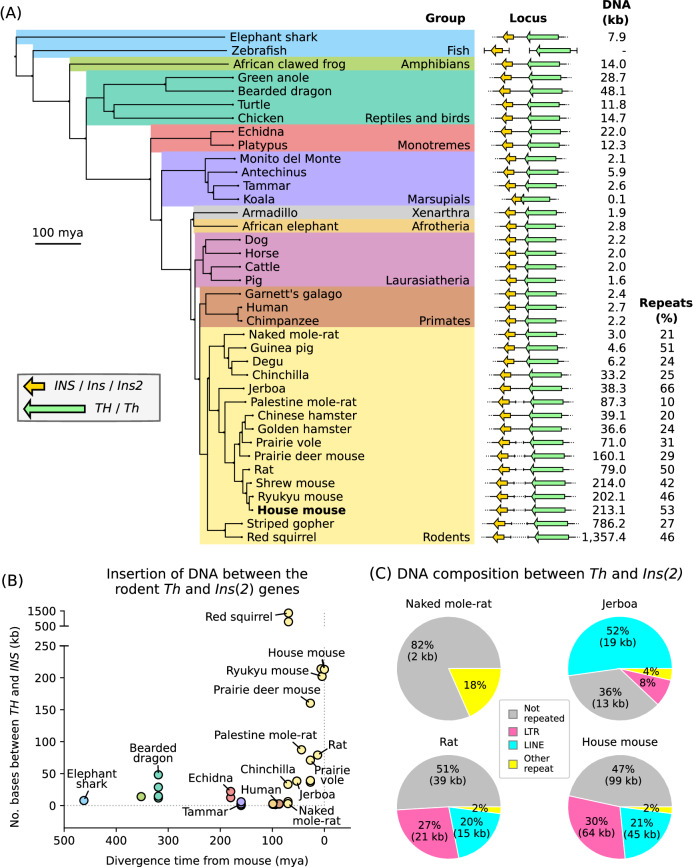
Accumulation of DNA between *Th* and *Ins(2)* orthologues over evolutionary time in the rodent lineage. **A** The number of bases between the *TH/Th* and *INS/Ins/Ins2* genes is given for different species and the relationships of those species are placed in terms of either their phylogenetic grouping or (**B**) the species divergence relative to the house mouse. Divergence times, millions of years ago (mya), are those indicated by TimeTree. Illustration of the gene locus (**A**) is not to scale. **C** The percent of the rodent DNA segment between *Th* and *Ins(2)* comprised of different classes of repeated elements is shown for the naked mole-rat, jerboa, rat and house mouse.

The DNA sequence between the orthologous *TH* and *INS* genes was assessed across 38 vertebrate species from diverse taxonomic groups (Fig. 1A). Across non-rodent therian species the length of the DNA segment between *TH* and *INS* was 2.4 kb with a standard deviation of 1.3 kb. In the fish, amphibians and reptiles that were assessed the DNA segment was 20 ± 13 kb, or linkage of the two genes was not maintained as was the case in the teleost zebrafish which had *th* on chr25 and *ins* on chr5. In the rodent lineage the DNA between *Th* and *Ins(2)* was highly variable in length ranging from 3 kb in the naked mole-rat (*Heterocephalus glaber*) to 1357 kb in the red squirrel (*Sciurus vulgaris*). The house mouse (*Mus musculus*) was similar to other species of mice and had a large 213.1 kb DNA segment between the *Th* and *Ins2* genes.

There was a progressive extension of the DNA segment between the *Th* and *Ins2* genes in rodents (Fig. 1B). The Hystricognathi clade, including the naked mole-rat and degu (*Octodon degus*), shared an ancestor with the murine rodents (Muroidea), including the house mouse, 70 mya and, with the exception of the chinchilla (*Chinchilla lanigera*), had a short DNA segment that was comparable to non-rodent therians. The jerboa (*Jaculus jaculus*) and the prairie vole (*Microtus ochrogaster*) diverged from mice 53 and 27 mya and had DNA segments that were 38 and 71 kb long respectively. The rat (*Rattus norvegicus*) shared an ancestor with the house mouse 13 mya and had 79 kb of DNA between *Th* and *Ins2*, reflecting a recent rapid extension in the length of the DNA segment in mice. The squirrel-related clade, including the red squirrel and striped gopher (*Ictidomys tridecemlineatus*), was an exception to this progression diverging from mice 69 mya yet having a DNA segment longer than 785 kb, suggesting a distinct evolutionary process in the squirrel lineage.

To gain insight into the DNA separating *Th* and *Ins(2)* in rodents we profiled the repeated element composition (Fig. 1A, C). Across the rodent species assessed, repeated elements comprised 35 ± 15% of the DNA segment. The amount of repeated DNA progressively increased between the *Th* and *Ins(2)* genes in the mouse-related clade. The jerboa which diverged before Muroidea had a distinct accumulation of long interspersed nuclear elements (LINEs) that made up 52% of the DNA between *Th* and *Ins* in this species. In Muroidea long terminal repeats (LTRs) were the largest subclass of repeated elements found. In the house mouse LTRs made up 30%, or 63.9 kb, of the DNA between the *Th* and *Ins2* genes. The most common individual LTR was *MYSERV-int*: a murine-specific endogenous retroelement which alone made up 6%, or 7 kb, of the house mouse DNA segment, the *MYSERV6-int* element contributed an additional 6.1 kb. LTRs were also prominent in the squirrel-related clade, making up 450.7 kb of the DNA segment in the red squirrel.

**Fig. 2:**
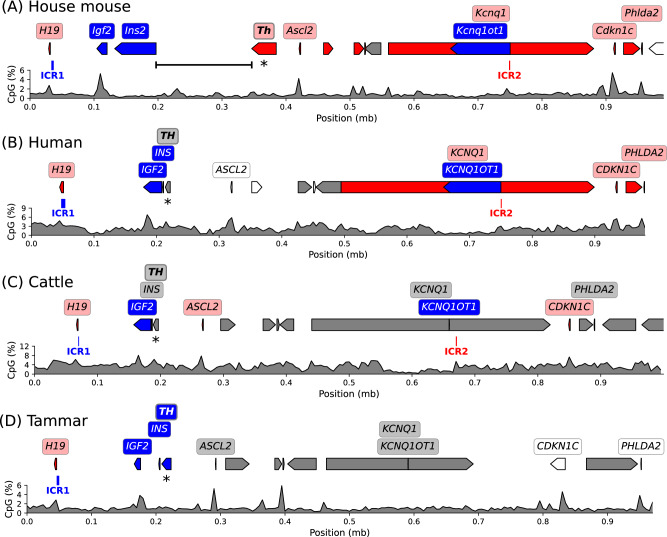
Parent-of-origin-specific expression in the broader ICR1/ICR2 imprinted region. The genomic context of the orthologous *TH/INS* locus is shown for the (**A**) house mouse, (**B**) human, (**C**) cattle and (**D**) tammar wallaby. The colour of the gene indicates parental expression; blue is paternal, red is maternal, grey is unknown, white is biallelic. The paternally-methylated ICR1 position is indicated in blue, the maternally-methylated ICR2 position is noted in red. The *TH* gene is marked with an asterisk. The genomic distance between *Th* and *Ins2* is indicated for the mouse (**A**). The *KCNQ1OT1* position in cattle and tammar indicates 500 and 400 bp amplicons that have been detected for this transcript (Ager et al. 2007; Robbins et al. 2012). Plots below display CpG density as a percentage averaged over 5 kilobase (kb) windows, mb: million base pairs.

To put the mouse *Th*/*Ins2* locus in the context of imprinting regulation, the gene positions were visualised to scale in the broader region (Fig. 2). In the house mouse the start site of the paternally-expressed *Ins2* gene was closer to ICR1 while the start site of the maternally-expressed *Th* gene was positioned halfway (48.9% of the distance) between ICR1 and ICR2 (Fig. 2A). In humans and cattle, species for which ICR positions are known, the start site of *TH* gene was positioned closer to ICR1, less than a quarter of the way between ICR1 and ICR2 (Fig. 2B, C). Parent-of-origin-specific expression for genes within the ICR1/ICR2 region has been best characterised in the house mouse with the imprinting status of *TH*, and multiple other genes, unknown in other species. Here we find, below, that *TH* is paternally-expressed in the tammar wallaby; though no ICR2 has been found, the position of *TH* relative to ICR1 in tammar wallabies resembles humans and cattle (Fig. 2D).

### The tammar wallaby chimaeric *TH-INS* transcript is paternally-expressed

**Fig. 3:**
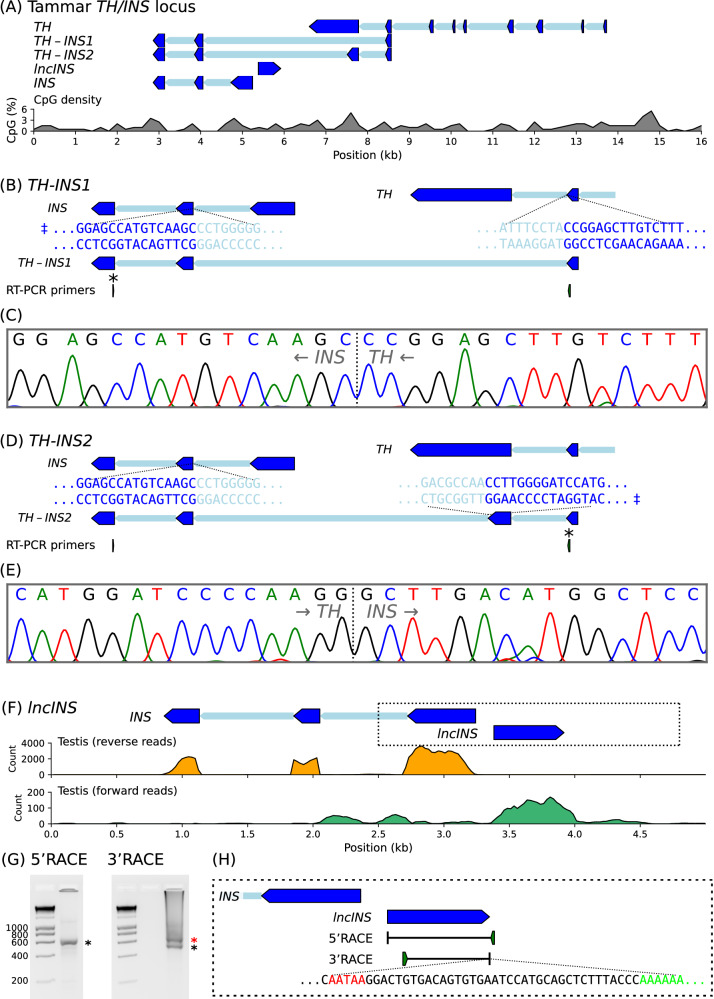
Organisation of the tammar wallaby *TH/INS* locus. **A** The tammar wallaby *TH/INS* gene locus showing exons as arrowheads indicating the direction of transcription, introns are thinner shaded regions. The blue colouring reflects the paternal expression pattern. Plot below displays CpG density as a percentage averaged over 200 bp windows. **B**, **D** Structure of the chimaeric *TH-INS1* and *TH-INS2* transcripts noting exonic (dark blue) and intronic (light blue) DNA sequences for the *TH* exon used and the second *INS* exon. An asterisk indicates the primer used for sequencing of the RT-PCR product and the double dagger indicates the sequence strand assessed. **C**, **E** Sanger sequencing chromatogram showing the junction, indicated by a dotted line, between the *TH* sequence and the *INS* sequence. Arrows indicate the direction of transcription. **F** Identification of an antisense transcript at the *INS* start site (dotted box) in testis transcriptome data with reads split into forward strand (green) and reverse strand (orange). Amplification of the antisense lncRNA was performed by (**G**) 5′ and 3′ RACE using adult testis cDNA. One of the two 3′RACE products (black asterisks) was isolated which encoded (**H**) a non-coding transcript with polyadenylation signal (red) and poly-A tail (green). The sequence of the larger 3′RACE product (red asterisks) was not confirmed.

**Fig. 4:**
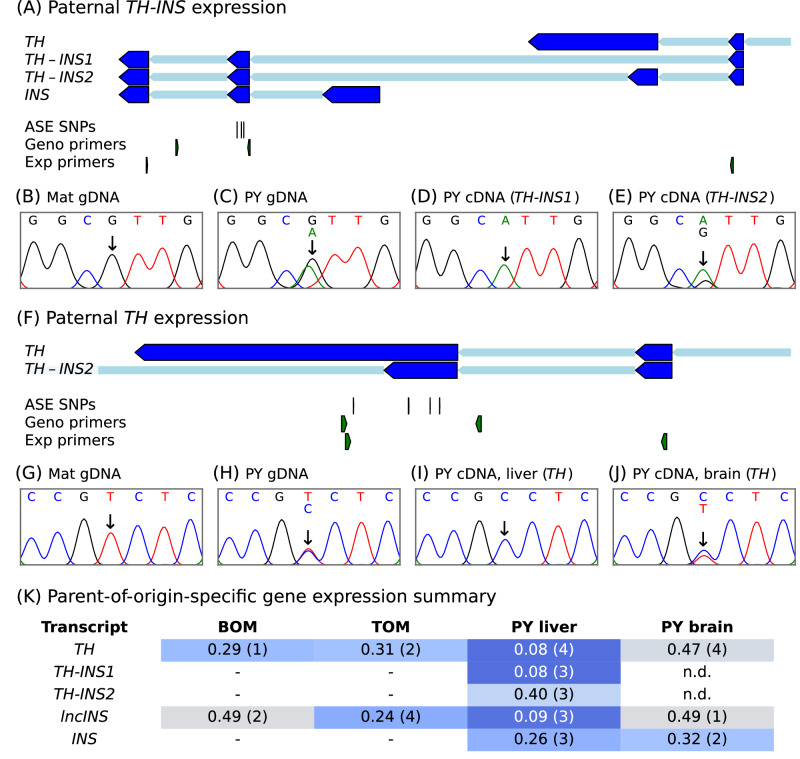
Paternal transcription of *TH/INS* in the tammar wallaby. **A**, **F** Primer and SNP positions for allele-specific expression (ASE) analysis of *TH-INS* and *TH*. The blue colour of the exons reflects the paternal expression pattern. The *TH-INS* genotyping primer set (Geno primers) had at least one intronic primer; the cDNA expression primer set (Exp primers) had an intron-spanning primer. **B**–**E**, **G**–**J** Parent-of-origin-specific transcription of *TH-INS* and *TH*. Sanger sequencing chromatograms showing the presence of (**B**, **G**) the homozygous maternal genotype, (**C**, **H**) the heterozygous SNP in PY gDNA, and (**D**, **E**, **I**, **J**) the allele present in PY cDNA. **K** Tabular summary of parent-of-origin-specific gene expression for the different RNA species detected from the *TH*/*INS* locus. A mean “mat:pat ratio” value closer to 0 indicates paternal expression (blue shading) while values closer to 0.5 indicate biallelic expression (grey shading). The number of animals is in brackets. “n.d.” indicates not detected. Placenta tissues taken from fetal stage samples, liver and brain taken from PY stage samples. BOM bilaminar omphalopleure, TOM trilaminar omphalopleure. See also, Supplementary Fig. 1 and Supplementary Table 3.

The close physical association between the *TH* and *INS* genes at the tammar wallaby locus (Fig. 3A) is exemplified by the production of a previously observed chimaeric *TH-INS* transcript (Stringer et al. 2012, 2014). RT-PCR, using an *INS* primer and a *TH* primer (Figs. 3B, D and 4A), resulted in two products that differed in size by 274 bp; these transcripts are referred to here as *TH-INS1* (GenBank: PP646883) and *TH-INS2* (GenBank: PP646884), where the latter includes part of the final exon of the *TH* gene. Analysis of the DNA sequence in the RT-PCR products showed the presence of a chimaeric junction between *TH* and *INS* sequences (Fig. 3C, E). When appropriate we refer to *TH-INS1* and *TH-INS*2 collectively as *TH-INS*.

To test whether *TH-INS* showed allele-specific expression we genotyped 23 PY for which liver and brain tissue was available and identified three informative animals with SNPs in an *INS* exon (Fig. 4B). Transcription of *TH-INS* was tissue-specific, with the *TH-INS1* and *TH-INS2* transcripts detected in PY liver, but not brain tissue. Allele-specific expression analysis (Fig. 4A–E) showed monoallelic paternal transcription of *TH-INS1* and skewed paternal transcription of *TH-INS2* in the PY liver. *TH-INS1* expression was from the paternal allele (Fig. 4K) in the three animals assessed, where the mat:pat ratio values were 0.01, 0.20 and 0.04. *TH-INS2* expression was variable, in the three animals assessed the mat:pat ratios values were 0.18, 0.28 and 0.75. As we expected from previous studies (Agar et al. 2008, Stringer et al. 2012) the *INS* gene itself (Fig. 4L, Supplementary Fig. 1F–J) had paternally-skewed expression across three PY livers and had paternally-skewed expression in two PY brain samples (mat:pat ratio values 0.34 and 0.30).

### *lncINS*: a new paternally-expressed long noncoding RNA antisense to *INS*

In eutherians, some imprinted genes are associated with long non-coding RNAs (lncRNAs): *H19*, *Kcnq1ot1* and *Airn* (Wan and Bartolomei 2008). Since *INS* and the associated chimaeric transcript *TH-INS1* showed paternal expression, it is possible that there is another lncRNA which regulates the coordinated imprinted expression, as seen in mouse *Kcnq1ot1*. In searching for additional elements relevant to the regulation of the *INS* locus, a new transcript was found (Fig. 3F–H). This 603 bp transcript (GenBank: PP646882) was discovered in a tammar wallaby adult testis transcriptome dataset (NCBI SRA: DRX012262). This RNA was transcribed from a position proximal to the *INS* start site in the antisense orientation (Fig. 3F). The Coding Potential Calculator 2 (Kang et al. 2017) gave this transcript a protein coding probability of less than 1%. LncRNAs are defined as RNAs longer than 200 nucleotides that are not translated into functional proteins (Statello et al. 2021), here this lncRNA is referred to as *lncINS*.

Genotyping for SNPs in *lncINS* identified six informative pre-natal animals of which four were assessed and three informative postnatal animals, out of 12 tested. Allele-specific expression analysis showed *lncINS* to be monoallelically expressed from the paternal allele in the PY liver (Fig. 4K, Supplementary Fig. 1K–S) with a mat:pat ratio of 0.09 ± 0.03 (*n* = 3 animals). In placenta *lncINS* expression was paternally-skewed in the TOM with a mat:pat ratio of 0.24 ± 0.04 (*n* = 4 animals) whilst in BOM expression was biallelic with a mat:pat ratio of 0.53 and 0.44 in *n* = 2 animals (Fig. 3K, Supplementary Fig. 1K–S). Parent-of-origin-specific expression of *lncINS* was particularly apparent in the TOM tissue of one fetus and the liver tissue of a PY, these animals both had five informative SNPs in *lncINS* that all were all preferentially expressed from the paternal allele (Supplementary Table 3). Further evidence for parent-of-origin-specific, but not allele-specific, expression of *lncINS* was illustrated by a C/T SNP site in which the T allele was maternally provided to a fetus that had skewed expression of the paternal C allele in the TOM and a PY that inherited a maternal C allele and expressed the paternal T allele in the liver (Supplementary Fig. 1L–S).

### The tammar wallaby has paternal-expression of *TH*

Since *INS*, *lncINS* and *TH-INS* had paternal expression, it was possible that imprinting might extend to the marsupial *TH* gene. We examined the allele-specific expression of the *TH* gene which is maternally-expressed in mice (Golding et al. 2011; Jones et al. 2011; Okae et al. 2012; Bonthuis et al. 2015). Genotyping for SNPs in *TH* identified five informative fetuses of which two were assessed and four informative PYs. Allele-specific expression analysis showed *TH* was also paternally-expressed (Fig. 3F–K). Paternal transcription of *TH* was monoallelic in the PY liver (mat:pat ratio of 0.08 ± 0.08, *n* = 4 animals), and paternally-skewed in both BOM and TOM placental tissue (Supplementary Fig. 1A–E). Providing further evidence for parent-of-origin-specific expression of *TH*, one of the SNP sites (C/T) showed inheritance of a maternal C allele in one animal and inheritance of a maternal T allele in another animal with the paternal allele expressed in the PY liver in both cases (SNP loc. 11, Supplementary Table 3). In contrast, *TH* expression was biallelic in the PY brain, with a mat:pat ratio for the four animals being 0.39, 0.39, 0.47 and 0.62.

## Discussion

The *TH* gene is expressed from the paternal allele in the tammar wallaby but from the maternal allele in the mouse. Changes in the parental origins of gene expression are rare. Of the 63 imprinted genes in common between human and mouse, differences in which allele is expressed have only been noted for Wilms’ tumour 1 (*Wt1*), bladder cancer-associated protein (*Blcap*), and zinc finger imprinted 2 (*Zim2*) (Tucci et al. 2019). *Zim2* may be the best example of a change in the parental origins of transcription between species. In humans *ZIM2* is paternally-expressed (Murphy et al. 2001), whereas in mice *Zim2* is maternally-expressed (Kim et al. 2004) like *TH*. *ZIM2* is located downstream of paternally expressed gene 3 (*PEG3*) at a similar distance in humans and in mice (Kim et al. 2004). So far no marsupial orthologue has been found for *PEG3* or *ZIM2* (Suzuki et al. 2011; Stringer et al. 2014). The change in the imprinting status of *Zim2* in the mouse appears to have resulted from an insertional event that placed the *Zim1* gene between *Peg3* and *Zim2* (Kim et al. 2004).

The large distance between mouse *Th* and *Ins2* is not the ancestral state of mammals but instead reflects the unique evolutionary path of rodents. The mouse genome is 36.5% transposon-derived and has a high activity of transposable elements, particularly LINEs (Mouse Genome Sequencing Consortium 2002; Thybert et al. 2018). The activity of transposable elements has varied over time in the primate and rodent lineages, with Muridae showing a recent expansion of LINEs (Thybert et al. 2018). While LINEs are present in the expanded region between the rodent *Th* and *Ins(2)* genes, particularly in the jerboa, the majority of the DNA separating these genes in mice is made up of LTRs (Shirohzu et al. 2004; Lefebvre et al. 2009). Though the function of the prominent *MYSERV6-int* element in this region is unclear, this element is environmentally responsive, and is upregulated in fetal mouse testicular cells after exposure of pregnant mothers to the obesogen tributyltin (Shioda et al. 2022).

The repeat-rich region between *Th* and *Ins2* in the mice may serve as a boundary between imprinted domains (Shirohzu et al. 2004). But, despite its size, this region does not pose a barrier to imprinting. In the mouse, the silencing effect of the *Kcnq1ot1* lncRNA extends from ICR2 some 470 kb, across the genetic region separating *Th* and *Ins2* (Jones and Lefebvre 2009). An isoform of *Ins2*, *Ins2-006*, is regulated by *Kcnq1ot1* and maternally-expressed in the E13.5 embryonic mouse head from an alternative promoter 20 kb upstream of the main *Ins2* gene (Jones et al. 2011). It is not known if *Th-Ins2* chimaeric transcripts are produced in the mouse. If a barrier is present between ICR1 and ICR2 then in mice that barrier should be positioned close to *Ins2*, between the alternative promoter that drives expression from the maternal isoform and the main promoter from which the paternal *Ins2* gene is transcribed (Golding et al. 2011).

Variation in the size of the DNA segment separating the *INS* and *TH* genes can have phenotypic consequences in humans. A polymorphic region comprising of a variable number of tandem repeats (VNTR) of a 14–15 nucleotide sequence is positioned upstream of the *INS* gene start site (Bennett and Todd 1996). The VNTR alleles occur in three size classes; class III (141–209 repeats) alleles are the largest and have a DNA segment at least 1 kb larger than the class I (26-63 repeats) alleles (Bennett and Todd 1996). In addition, a tetranucleotide microsatellite, *TH01*, is located within the *TH* gene and can affect gene expression (Berumen 2023). The class III allele is associated with protection against type 1 diabetes but susceptibility to type 2 diabetes when paternally derived (Bennett and Todd 1996; Huxtable et al. 2000). In the presence of the autoimmune regulator, AIRE, the class III VNTR locus drives expression of insulin at levels three-fold higher than the class I locus in thymic endothelial cells (Cai et al. 2011).

There are multiple modes of monoallelic expression (Reinius and Sandberg 2015) and we conclude that the tammar wallaby *TH/INS* gene locus has parent-of-origin-specific gene expression from the paternal allele. Paternal expression of the *TH*/*INS* region may result from differential methylation at the distal ICR1 site, the imprint associated with *H19* and *IGF2*. The imprinting effect of ICR1 could extend to the *TH/INS* region. Binding of the insulator CTCF to the *H19* DMR on the maternal allele changes the chromatin architecture such that *IGF2*, the gene adjacent to *INS*, is maternally-silenced (Smits et al. 2008; Hore et al. 2010; Llères et al. 2019). In mice, *H19* and *Igf2* are imprinted in both embryonic and extraembryonic tissues while paternal expression of *Ins2* is restricted to the yolk sac (Deltour et al. 1995, 2004; Hudson et al. 2010).

We expanded the list of transcripts from the marsupial *TH/INS* locus to include *TH*, *TH-INS1*, *TH-INS2*, *lncINS*, and *INS*. Imprints are often associated with lncRNAs that can regulate gene expression through various mechanisms (Autuoro et al. 2014; Statello et al. 2021), the antisense lncRNA is typically expressed from a different parental origin but *lncINS* is expressed from the same parental origin as *INS*. The novel marsupial *lncINS* is not as long as other imprinted lncRNAs in eutherians, this was similar to the marsupial antisense lncRNA in the *IGF2R* DMR, *ALID* (Suzuki et al. 2018). The degree of paternal transcription was stronger in the PY liver, implying the locus had undergone broader maternal silencing in this tissue. In placental tissue and the PY brain, transcripts from the *TH/INS* locus showed a mix of biallelic and paternally skewed expression suggesting that regions of the domain are individually regulated.

The levels of transcript expression from the *TH/INS* locus were not assessed. The abundance of a transcript does not provide any indication of its functional importance, for example lncRNAs are important cellular regulators that are often found at low levels (Seiler et al. 2017; Grammatikakis 2022). The promiscuity of RNA polymerase can result in “spurious transcription”: a low background level of genome-wide transcription, even of the non-coding DNA (Jensen et al. 2013; Wade and Grainger 2018). The structure of the transcripts assessed from the *TH/INS* locus is much more organised than would be expected of spurious transcripts. Detection of known tissue-specific transcripts at low abundance in non-specific cells, without any likely role, has been referred to as “illegitimate transcription” (Chelly et al. 1989). Low transcript abundance could make allele-specific expression analysis more subject to gene-intrinsic noise (Kærn et al. 2005), but the transcripts from the *TH/INS* locus had consistent paternal monoallelic expression.

It is unlikely that the *TH-INS* transcripts are products of transcription termination failure. The chimaeric tammar *TH-INS* transcripts have a structure consistent with the most frequent form of *cis*-SAGe (*cis*-splicing of adjacent gene) chimaeras which are formed by the uninterrupted transcription of two nearby genes in the same orientation joining the second-to-last exon of the 5′ gene to the second exon of the 3′ gene (Chwalenia et al. 2017). Several mechanistic models have been proposed for how the transcriptional termination signal might be omitted from the 5′ gene, including; mutation of the polyA signal, torsional stress and “transcriptional slippage” (Chwalenia et al. 2017). The two parental genes that generate a fusion transcript typically have a small intergenic distance (Varley et al. 2014). The association of the *TH* and *INS* genes in many lineages may have been conserved to allow transcriptional read-through from *TH* to *INS* (Hernández-Sánchez et al. 2006).

**Fig. 5:**
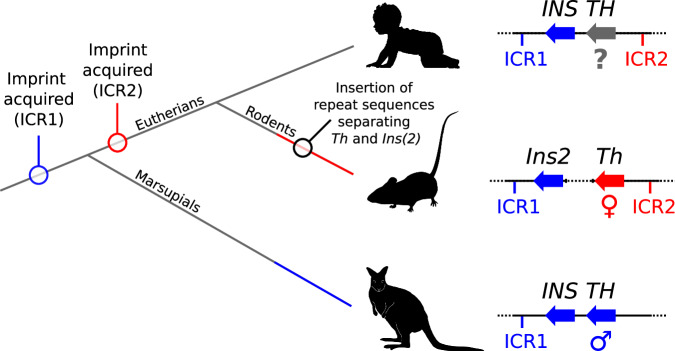
The possible evolutionary path leading to a change in the parental origins of *TH/Th* expression. (Left) Cladogram indicating the acquisition of ICR1 in the therian ancestor, acquisition of ICR2 in the eutherian ancestor and the separation of *Th* and *Ins(2)* in the rodent lineage. Maternal *Th* expression in the rodent lineage is indicated by the branch having a red colour. Paternal *TH* expression in the marsupial lineage is indicated by the branch having a blue colour. The unknown parent-of-origin-specific expression of *TH* in non-rodent eutherians and ancestral therians is indicated by the grey line colour. (Right) Simplified diagram of the *TH/INS* region highlighting the unknown parental origin of expression (grey) of *TH* in non-rodent eutherians, maternal expression (red) of *Th* in the mouse and paternal expression (blue) of *TH* in the wallaby. Also indicated is the acquisition of the paternally-methylated (blue) ICR1 and maternally-methylated (red) ICR2. The larger distance between *Th* and *Ins2* in the mouse is indicated by a dotted line between the genes. Species silhouettes include *Homo sapiens sapiens* by Andrew A. Farke (CC BY 3.0), *Murinae* by Katy Lawler (CC BY 4.0) and *Notamacropus eugenii* by Geoff Shaw (CC BY 4.0), courtesy of https://www.phylopic.org.

As the *TH* gene can vary between species in its parental origin of expression (Fig. 5), *TH* could be an “innocent bystander” to the effects of local gene regulation (Duvillié et al. 1998; Barlow and Bartolomei 2014). If the parental origin of *TH* expression correlated with the length of the DNA region between the *TH* and *INS* genes then all rodents that have inserted repeat sequences separating *Th* and *Ins2* would have maternal expression of *Th* and all non-rodent therians that have a close physical association of the *TH* and *INS* genes would have paternal expression of *TH*. However, the imprinting status of the *KCNQ1OT1* lncRNA is another important variable.

Maternal expression of *Th* could be rodent-specific. Placenta-specific imprinted *Th* isoforms in the mouse are promoted from a rodent LTR located between *Th* and the adjacent achaete-scute complex homologue 2 gene, *Ascl2* (Jones et al. 2011). *Ascl2* is also maternally-expressed in the mouse placenta (Guillemot et al. 1995), but *ASCL2* is biallelically-expressed in the human placenta from 12 to 39 weeks gestation (Miyamoto et al. 2002). A lack of imprinting at *ASCL2* and other neighbouring genes, such as *CD81*, in humans could mean that the domain of paternal-silencing by *KCNQ1OT1* is shorter in humans, relative to mice, potentially allowing for paternal expression of *TH*. However, mouse extraembryonic stem cells have maternal expression of *Th* and biallelic *Ascl2*, so it is possible for *ASCL2* to be either included or excluded from repression of the paternal locus (Golding et al. 2011).

The *TH* and *INS* genes lie at the interface of two imprints implicated in developmental pathology. In humans, and other non-rodent therians, whether *TH* is imprinted is unknown and few studies of *INS* imprinting have been performed. Mice are commonly used to model the Beckwith–Wiedemann and Silver–Russell syndromes, but some genomic features in this region are specific to rodents. As the mouse and tammar wallaby express *TH* from different parental alleles, it would be informative to assess parent-of-origin-specific expression in non-rodent therians. Our findings raise the possibility that paternal expression of *TH* was the ancestral condition in therians (Fig. 5) with the parental origins of expression changing in either the eutherian lineage with acquisition of the maternally methylated ICR2 or more recently in the rodent lineage following the extensive insertion of repeat elements between *TH* and *INS*.

### Supplementary information

Supplementary information

## Data Availability

The analysed datasets are available, further information provided in Supplementary Table 1. Sequences for lncINS (PP646882), TH-INS1 (PP646883), TH-INS2 (PP646884) are available at GenBank (https://www.ncbi.nlm.nih.gov/genbank/).
