# Supplementary material for: The structure of the TH/INS locus and the parental allele expressed are not conserved between mammals
Source: Heredity (Edinb). 2024 Jun 4;133(1):21–32. doi: 10.1038/s41437-024-00689-y (PMC11222543; doi:10.1038/s41437-024-00689-y)
Supplement: Supplementary file 1 — Supplementary information [file 41437_2024_689_MOESM1_ESM.pdf]

Supplementary information

Name	Assembly	Accession	*TH*-to-*INS* (bp)	Mouse diverge (mya)
African clawed frog	Xenopus_laevis _v10.1	GCA_017654675.1	13953	352
African elephant	Loxafr3.0	GCA_000001905.1	2831	99
Antechinus	AdamAnt_v2	GCA_016432865.2	5902	160
Armadillo	mDasNov1.hap2	GCA_030445035.1	1876	99
Bearded dragon	pvi1.1	GCA_900067755.1	48137	319
Cattle	ARS-UCD2.0	GCA_002263795.4	1977	94
Chicken	bGalGal1.mat .broiler.GRCg7b	GCA_016699485.1	14733	319
Chimpanzee	NHGRI_mPanTro3-v1.1-hic.freeze_pri	GCA_028858775.1	2250	87
Chinchilla	ChiLan1.0	GCA_000276665.1	33171	70
Chinese hamster	CriGri_1.0	GCA_000223135.1	39138	26
Degu	OctDeg1.0	GCA_000260255.1	6176	70
Dog	UU_Cfam_GSD_1.0	GCA_011100685.1	2203	94
Echidna	mTacAcu1	GCA_015852505.1	22011	180
Elephant shark	IMCB_Cmil_1.0	GCA_018977255.1	7852	462
Garnett's galago	OtoGar3	GCA_000181295.3	2390	87
Golden hamster	BCM_Maur_2.0	GCA_017639785.1	36627	26
Green anole	AnoCar2.0	GCA_000090745.2	28715	319
Guinea pig	Cavpor3.0	GCA_000151735.1	4586	70
Horse	EquCab3.0	GCA_002863925.1	2002	94
House mouse	GRCm39	GCA_000001635.9	213053	0
Human	GRCh38.p14	GCA_000001405.29	2720	87
Jerboa	mJacJac1.mat.Y.cur	GCA_020740685.1	38279	53
Koala	phaCin_unsw_v4.1	GCA_002099425.1	90	160
Monito del Monte	mDroGli1	GCA_019393635.1	2089	160
Naked mole-rat	HetGla_female_1.0	GCA_000247695.1	3038	70
Palestine mole-rat	S.galili_v1.0	GCA_000622305.1	87272	44
Pig	Sscrofa11.1	GCA_000003025.6	1576	94
Platypus	mOrnAna1.pri.v4	GCA_004115215.4	12343	180
Prairie deer mouse	HU_Pman_2.1.3	GCA_003704035.3	160130	26
Prairie vole	MicOch1.0	GCA_000317375.1	70990	26
Rat	mRatBN7.2	GCA_015227675.2	78978	13
Red squirrel	mSciVul1.1	GCA_902686455.1	1357392	69
Ryukyu mouse	CAROLI_EIJ_v1.1	GCA_900094665.2	202130	4
Shrew mouse	PAHARI_EIJ_v1.1	GCA_900095145.2	213968	6
Striped gopher	HiC_Itri_2	GCA_016881025.1	786210	69
Tammar	MeugV7.0	-	2649	160
Turtle	Chrysemys_picta _BioNano-3.0.4	GCA_000241765.5	11841	319
Zebrafish	GRCz11	GCA_000002035.4	13780195	429

Supplementary Table 1. Genomes used for the species comparison of *TH* and *INS* separation. Divergence times from the house mouse provided by TimeTree 5.

Name	Sequence	Use	Reference
TH-INS_primer_F	CCAGAGCCTTCTGGGGACCT	Geno	This study
TH-INS_primer_R	CTCCCTAGTGGCCCTGCTCA	Geno	This study
RvIntron-Ins	GCAGGGAGAGGACAGGAAATC	Geno	Ager 2007
Fw3UTR-Ins	CTACCCCCAGGGCTTGACATG	Geno	Ager 2007
TH-INS_F01	TTCGTGTCAGAGAGCTTTAGCGATG	Exp	Stringer 2012
TH-INS_R01	TCCGGCCTCACCCAACAGTG	Exp	Stringer 2012
INS_cDNA_Fw	GCTCCATCTTCTTTTCTCACCTGTCTCT	Exp	Stringer 2012
Q3INS_Rv	TGGCCGCTAGTTGCAGTAAGTC	Exp	Stringer 2012
lncINS-TH_F01	CCCTGGGCCTAGATACATTGGAGGA	Geno/Exp/ 3’RACE	This study
lncINS-TH_R01	GTGTAGTTCGGAAGCAGCCTCAGAG	Geno/Exp	This study
lncINS-TH_R02	CAGAGAGAACCAAACAGGATGGCGT	5’RACE	This study
MeTH_3utr_g_1_F	GACATGCCATAAGGGTCAGG	Geno	This study
MeTH_3utr_g_1_R	GGTCTGCTCTGGGACACTTC	Geno	This study
MeTH_3utr_c_1_F	GTCAGGGCAGGGATCTCAAT	Exp	This study
MeTH_3utr_c_1_R	CAGAGATCCGGGAGTTTGAC	Exp	This study

Supplementary Table 2. Primers used in this study. ”Geno”: genotyping primers, ”Expr”: intron-spanning expression primers.

Transcript	Tissue	Animal	SNP loc.	Major allele	Major signal	Minor allele	Minor signal	Mat. allele
TH	BOM	2	12	T	188	C	84	C
TH	BOM	2	13	A	505	G	197	G
TH	TOM	1	11	T	303	C	170	C
TH	TOM	2	12	T	347	C	131	C
TH	TOM	2	13	A	499	G	173	G
TH	PY liver	4	9	C	213	G	1	A
TH	PY liver	4	10	C	259	G	0	G
TH	PY liver	4	12	T	336	G	1	C
TH	PY liver	6	11	T	370	C	175	C
TH	PY liver	7	9	C	359	G	2	A
TH	PY liver	7	10	C	462	G	0	G
TH	PY liver	7	12	T	379	G	1	C
TH	PY liver	7	13	A	584	G	1	G
TH	PY liver	9	11	C	317	G	2	T
TH	PY brain	4	9	C	180	A	157	A
TH	PY brain	4	10	C	186	G	100	G
TH	PY brain	4	12	C	171	T	117	C
TH	PY brain	6	11	C	210	T	129	C
TH	PY brain	7	9	A	240	C	232	A
TH	PY brain	7	10	C	190	G	90	G
TH	PY brain	7	12	T	186	C	110	C
TH	PY brain	7	13	A	412	G	211	G
TH	PY brain	9	11	C	182	T	115	T
TH-INS1	PY liver	4	1	T	443	A	58	C
TH-INS1	PY liver	4	2	A	345	G	0	G
TH-INS1	PY liver	4	2	A	276	T	3	G
TH-INS1	PY liver	7	2	A	293	T	3	G
TH-INS1	PY liver	8	1	T	397	C	106	C
TH-INS2	PY liver	4	2	G	391	A	131	G
TH-INS2	PY liver	7	2	A	238	G	93	G
TH-INS2	PY liver	8	1	T	311	C	67	C
lncINS	BOM	2	4	T	303	C	240	C
lncINS	BOM	3	3	T	215	C	188	T
lncINS	TOM	1	3	T	235	C	112	C
lncINS	TOM	2	4	T	452	C	169	C
lncINS	TOM	3	3	C	269	T	78	T
lncINS	TOM	5	4	C	309	T	40	T
lncINS	TOM	5	5	A	685	G	36	G
lncINS	TOM	5	6	C	436	T	123	T
lncINS	TOM	5	7	A	218	G	23	G
lncINS	TOM	5	8	T	441	C	120	C
lncINS	PY liver	6	3	T	253	C	19	C
lncINS	PY liver	7	4	T	452	C	95	C
lncINS	PY liver	7	5	G	498	A	129	A
lncINS	PY liver	7	6	T	292	C	32	C
lncINS	PY liver	7	7	G	431	A	85	A
lncINS	PY liver	7	8	C	336	T	24	T
lncINS	PY liver	9	3	C	283	G	16	T
lncINS	PY brain	6	3	T	395	C	377	C
INS	PY liver	4	1	T	498	A	34	C
INS	PY liver	4	2	A	262	G	0	G
INS	PY liver	7	2	G	367	A	131	G
INS	PY liver	8	1	T	431	A	3	C
INS	PY brain	4	1	T	266	C	91	C
INS	PY brain	4	2	A	168	G	124	G
INS	PY brain	8	1	T	167	C	122	C

Supplementary Table 3. Allele-specific expression analysis. The chromatogram signals for the major and minor alleles, the maternal allele ("Mat. allele") indicates the parental origin. Replication is noted across particular animals and SNP locations ("SNP loc.").

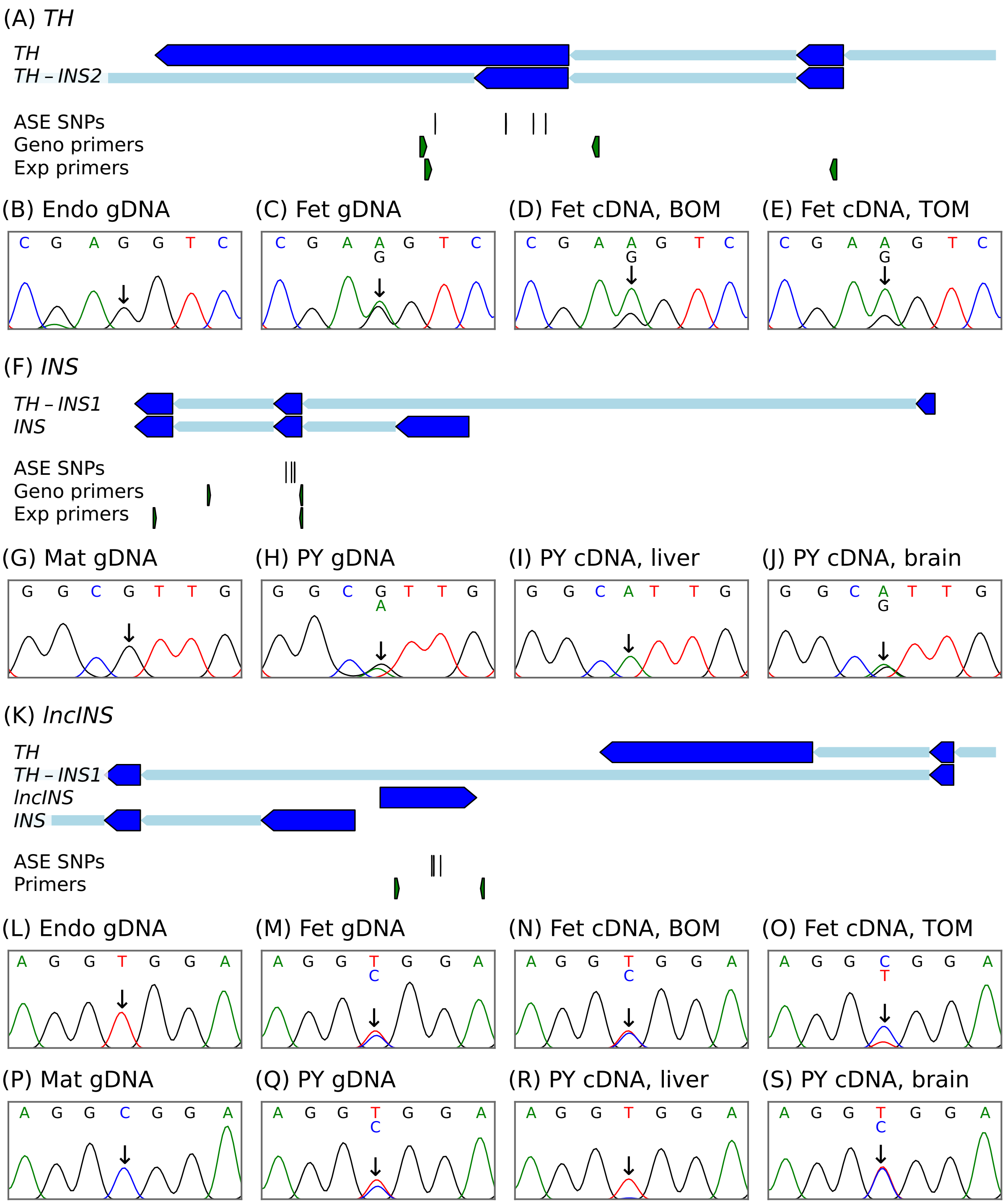

Supplementary Fig. 1. Paternal expression of the *TH/INS* locus. (A) Primer and SNP positions for allele-specific expression analysis of *TH*. The *TH* genotyping primer set (Geno primers) had an intronic primer; the cDNA expression primer set (Exp primers) had an intron-spanning primer in the *TH* exon. (B-E) Pre-natal paternally-skewed transcription of *TH* in placental tissue. Sanger sequencing chromatograms showing the presence of (B) the homozygous maternal genotype, (C) the heterozygous SNP in fetal (Fet) gDNA, (D) paternally-skewed expression of *TH* in BOM cDNA and (E) TOM cDNA. (F) Primer and SNP positions for allele-specific expression analysis of *INS*. (G-J) Tissue-specific post-natal paternal transcription of *INS*. Sanger sequencing chromatograms showing the presence of (G) the homozygous maternal genotype, (H) the heterozygous SNP in PY gDNA, (I) paternal expression of *INS* in PY liver cDNA and (J)

biallelic expression of *INS* in PY brain cDNA. (K) Primer and SNP positions for allele-specific expression analysis of *lncINS*. (L-O) Tissue-specific pre-natal paternal transcription of *lncINS* in placental tissue and (P-S) tissue-specific post-natal paternal transcription of *lncINS*. Sanger sequencing chromatograms showing the presence of (L) the homozygous maternal genotype in the endometrial (Endo) tissue, (M) the heterozygous SNP in fetal gDNA, (N) biallelic expression of *lncINS* in BOM cDNA and (O) paternally-skewed expression of *lncINS* in TOM cDNA. Sanger sequencing chromatograms showing the presence of (P) the homozygous maternal genotype, (Q) the heterozygous SNP in PY gDNA, (R) paternal expression of *lncINS* in PY liver cDNA and (S) biallelic expression of *lncINS* in PY brain cDNA.
